# Identification of genes and pathways associated with menopausal status in breast cancer patients using two algorithms

**DOI:** 10.1186/s12905-023-02846-7

**Published:** 2024-01-02

**Authors:** Minzhang Cheng, Lingchen Wang, Yanlu Xuan, Zhenyu Zhai

**Affiliations:** 1 Jiangxi Clinical Research Center for Respiratory Diseases, Jiangxi Institute of Respiratory Disease, the Department of Respiratory and Critical Care Medicine, The First Affiliated Hospital, Jiangxi Medical College, Nanchang University, Nanchang, Jiangxi 330006 China; 2 Jiangxi Key Laboratory of Molecular Diagnostics and Precision Medicine, Center for Experimental Medicine, The First Affiliated Hospital, Jiangxi Medical College, Nanchang University, Nanchang, Jiangxi 330006 China; 3 School of Public Health, University of Nevada, Reno, Reno, Nevada 89557 USA

**Keywords:** Breast cancer, Menopausal status, Differentially expressed genes, Signaling pathway

## Abstract

**Background:**

Menopausal status has a known relationship with the levels of estrogen, progesterone, and other sex hormones, potentially influencing the activity of ER, PR, and many other signaling pathways involved in the initiation and progression of breast cancer. However, the differences between premenopausal and postmenopausal breast cancer patients at the molecular level are unclear.

**Methods:**

We retrieved eight datasets from the Gene Expression Omnibus (GEO) database. Differentially expressed genes (DEGs) associated with menopausal status in breast cancer patients were identified using the MAMA and LIMMA methods. Based on these validated DEGs, we performed Gene Ontology (GO) functional enrichment and Kyoto Encyclopedia of Genes and Genomes (KEGG) pathway enrichment analyses. Protein–protein interaction (PPI) networks were constructed. We used DrugBank data to investigate which of these validated DEGs are targetable. Survival analysis was performed to explore the influence of these genes on breast cancer patient prognosis.

**Results:**

We identified 762 DEGs associated with menopausal status in breast cancer patients. PPI network analysis indicated that these genes are primarily involved in pathways such as the cell cycle, oocyte meiosis and progesterone-mediated oocyte maturation pathways. Notably, several genes played roles in multiple signaling pathways and were associated with patient survival. These genes were also observed to be targetable according to the DrugBank database.

**Conclusion:**

We identified DEGs associated with menopausal status in breast cancer patients. The association of these genes with several key pathways may promote understanding of the complex characterizations of breast cancer. Our findings offer valuable insights for developing new therapeutic strategies tailored to the menopausal status of breast cancer patients.

**Supplementary Information:**

The online version contains supplementary material available at 10.1186/s12905-023-02846-7.

## Introduction

As the leading cancer diagnosis in women, breast cancer accounted for approximately 2,261,000 new cases and 684,000 fatalities in 2020 [1]. This hormone-dependent malignancy primarily affects the mammary gland in females. Accurate identification of menopausal status is vital for effective prevention, detection, and treatment [2, 3]. A population-based study investigating the impact of premenopausal and postmenopausal breast cancer revealed that the mortality rate of patients with postmenopausal breast cancer in 2018 was 3.7 times greater than that in patients with premenopausal breast cancer [4]. Given the unique molecular characteristics of these two conditions, personalized strategies are required to manage breast cancer based on menopausal status. For instance, endocrine therapy, which reduces estrogen or progesterone levels, is recommended for postmenopausal patients with estrogen receptor (ER) or progesterone receptor (PR) positivity but is unsuitable for premenopausal patients [5, 6]. It has been widely recognized that menopausal status is associated with estrogen, progesterone, and other sex hormone levels, potentially influencing the activity of ER, PR and many other signaling pathways participating in the initiation and progression of breast cancer. However, the intricate molecular distinctions between premenopausal and postmenopausal breast cancer remain opaque. This gap in understanding impedes the full realization of precision medicine tailored to menopausal status. Therefore, enhancing our understanding of the unique molecular mechanisms of breast cancer through gene expression profile analyses is essential to improve early detection, diagnosis, and treatment strategies.

With the significant advancements in high-throughput technologies for genome-wide profiling of methylation events and gene expression levels, including methods such as methylation microarrays, MeDip-seq, and RNA-seq, and the availability of public datasets, we can now analyze data collected worldwide. Leveraging bioinformatic methods, we have the tools to identify potential biomarkers and pathways linked to menopausal status. However, numerous challenges arise in the integration and analysis of datasets from different sources. Fortunately, improvement in the differential expression analysis method enables us to perform cross-study analysis. In recent years, various differential expression analysis methods have been proposed, providing a variety of tools to ensure the robustness of our research findings.

To date, large-scale bioinformatic studies focusing on the differentially expressed genes (DEGs) associated with menopause in breast cancer patients have been scarce. The primary objective of our study is to illuminate the molecular distinctions between premenopausal and postmenopausal breast cancer patients. In our study, we attempted to collect more datasets to increase the sample size. In an integrated large cohort, we performed differential expression analyses to identify DEGs using two different algorithms. Additionally, Gene Ontology functional enrichment and Kyoto Encyclopedia of Genes and Genomes pathway enrichment analyses of the DEGs were performed. In addition, protein–protein interaction (PPI) networks were constructed to further elucidate the direct and indirect associations between the DEGs. In doing so, we hope to pinpoint key menopause-related biomarkers that could prove instrumental in future breast cancer research. Furthermore, understanding these biomarkers will undoubtedly shed light on the disease’s pathogenesis, offering new avenues for clinical drug development and therapeutic interventions.

## Methods

### Microarray data for differentially expressed gene (DEG) analysis

**Fig. 1:**
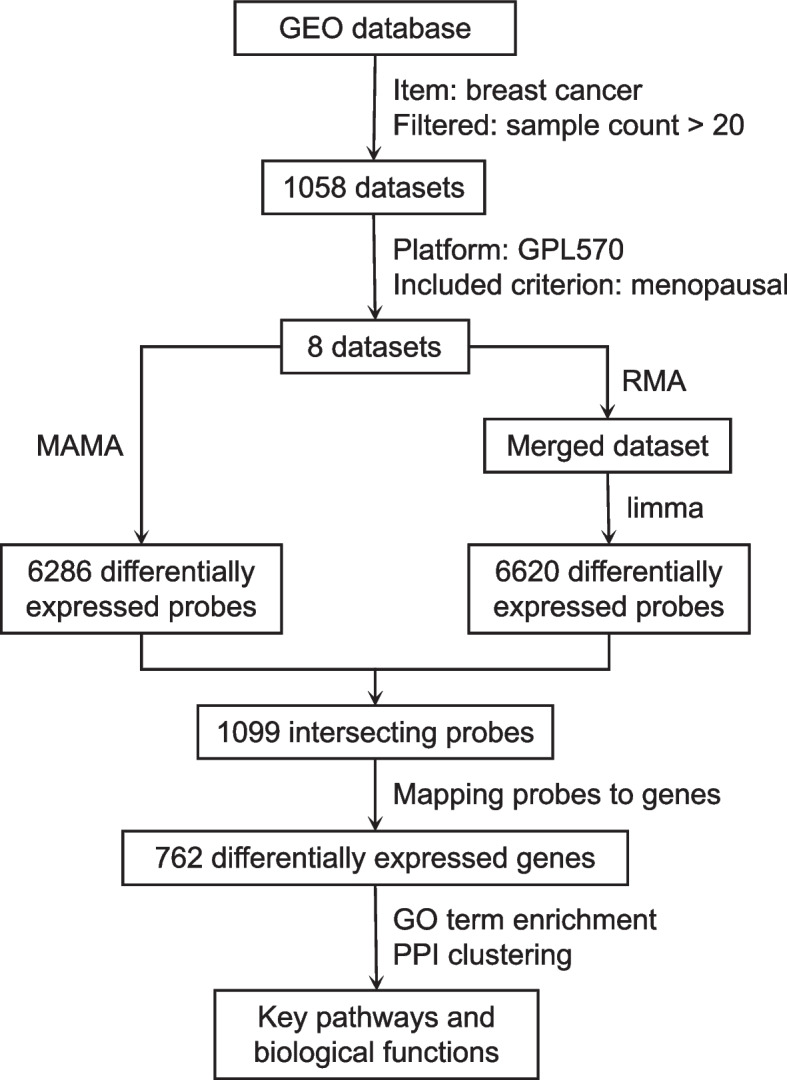
Process of screening genes and pathways associated with menopausal status in breast cancer patients using two algorithms

We conducted an extensive search for breast cancer microarray datasets with a sample size of more than 20 in the Gene Expression Omnibus (GEO) database from the National Center for Biotechnology Information (NCBI) website (https://www.ncbi.nlm.nih.gov/geo/). From the 1058 breast cancer datasets identified, we specifically selected datasets based on the Affymetrix Human Genome U133 Plus 2.0 Array (platform GPL570, *n* of probes = 54,675) that included primary breast cancer tissues (excluding cell lines or animal tissues) and had information on menopausal status. Raw intensity files in CEL format from these 8 selected datasets were obtained from the GEO database. The R package “affy” was employed to convert the raw intensity files to gene expression profiles using the robust multiarray average (RMA) method [7]. All data processing and statistical analyses were conducted in the R environment (https://www.rproject.org). The study process is graphically represented in Fig. 1.

### Differential expression analyses based on two algorithms

To identify DEGs, we utilized two different algorithms. First, we employed the R package “MAMA” to generate combined *p* values and combined effect sizes for the expression of each probe across all selected datasets [8]. The cutoff criteria for this method were set as a combined *p* value of less than 0.01 and a combined z score of greater than 2 or less than − 2. Then, all the samples from the 8 selected datasets were integrated into a large cohort. The R package “limma” was then used to calculate the adjusted *p* value and fold change for the expression of each probe in this integrated cohort [9]. The cutoff criteria for this method were an adjusted *p* value of less than 0.01 and a fold change (log 2) of greater than 2 or less than − 2. Probes demonstrating differential expression with consistent trends in both methods were selected for further analyses, and the genes mapped by these probes were identified as DEGs.

### Visualization of the expression of DEGs

To present the top 50 differentially expressed probes, we utilized the R package “pheatmap” to generate heatmaps. Given that the sample sizes varied across datasets, we randomly selected a subset of 50 samples for plotting. The heatmap employed unsupervised hierarchical clustering using the Ward method with Manhattan distance to visualize the clustering patterns of either the samples or probes. At the top of the heatmaps, the category of each selected sample (premenopausal or postmenopausal) was marked. We have uploaded the pipeline of differential expression analyses and visualization to GitHub. It can be accessed at https://github.com/minzhangcheng/BRCA_menopause.

### Enrichment analysis of GO terms and KEGG pathways

We utilized the WEB-based Gene SeT AnaLysis Toolkit (WebGestalt) for the enrichment analysis of Gene Ontology (GO) terms and Kyoto Encyclopedia of Genes and Genomes (KEGG) pathways [10, 11]. The overrepresentation analysis (ORA) method was employed, with a significance threshold of *p* values less than 0.05, to identify the critical biological implications of the DEGs.

To further illustrate the direct and indirect associations among the DEGs, protein–protein interaction (PPI) networks were constructed and visualized using the Search Tool for the Retrieval of Interacting Genes/Proteins (STRING) database [12]. The PPI networks were subsequently clustered using Cytoscope software [13] with the MCODE [14] plugin. Additionally, the setsApp [15] plugin was employed to color-code the network, highlighting gene sets associated with various clusters.

### Exploration of targeted compounds for and patient survival related to DEGs

Drugs and their targets were downloaded from the DrugBank database (https://go.drugbank.com/) to investigate the potential targetability of these validated DEGs [16]. Moreover, we utilized the TCGA breast cancer dataset to determine whether these genes influenced the overall survival (OS) of breast cancer patients.

## Results

### Dataset characteristics

Of the 1058 breast cancer datasets available in the GEO database, we selected 8 datasets that met our criteria for analysis. These datasets were GSE76124 [17], GSE43365 [18], GSE43502 [19], GSE50948 [20], GSE58792 [21], GSE71258 [22], GSE76274 [23] and GSE140494 [24]. These datasets contained 300 samples from premenopausal individuals and 393 samples from postmenopausal individuals (Table 1).

**Table 1:** Datasets involved in this study

Dataset	Sample counts		
	Total	Pre-menopausal	Post-menopausal
GSE76124	198^a^	62	94
GSE50948	156	72	84
GSE71258	128	59	41
GSE43365	111	32	67
GSE140494	91	45	44
GSE76274	67^a^	11	37
GSE58792	51	9	13
GSE43502	25^a^	10	13
Total	827^a^	300	393

^a^These datasets contain normal, unannotated, and samples that are not primary breast cancer. After excluding these samples, 693 samples that met our criteria were used for subsequent analyses

### DEGs associated with menopause in breast cancer patients

**Fig. 2:**
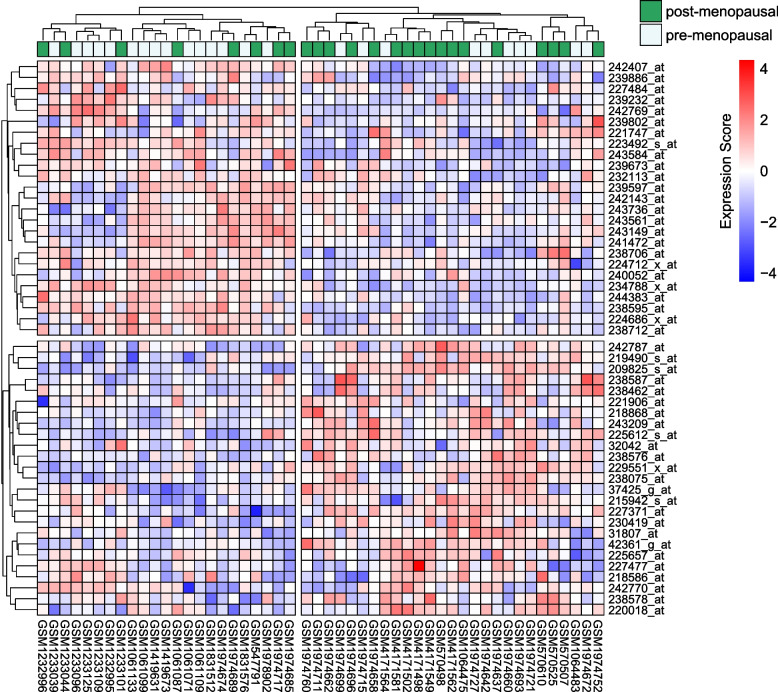
The expression profiles are presented in the heatmap of the top 50 DEGs in the integrated cohort. The expression levels of the genes are represented by different colors. Red, upregulated; Blue, downregulated. Each row represents a probe, and each column represents a sample

We used two distinct methods to screen for DEGs. In the first method, we treated the 8 datasets as separate cohorts. Within each dataset, we calculated *p* values and effect sizes and then generated combined *p* values and effect sizes. Applying a threshold of a combined *p* value less than 0.01 and a combined z score above 2, we identified 6286 differentially expressed probes (Table S1). In the second method, the samples from the 8 datasets were merged into a single large cohort. Using a cutoff of an adjusted *p* value less than 0.01 and a fold change (log2) greater than 2, 6620 probes were identified as differentially expressed probes (Table S1). The 1099 probes identified as differentially expressed by both methods were selected for further analyses and were found to map to 762 genes (Fig. 1, top 100 probes in Table 2, full list in Table S1). Additionally, heatmaps displaying the expression levels of the top 50 validated probes are presented in Fig. 2.

**Table 2:** Top 100 different expression probes

	Symbol	MAMA		limma	
		*p*	zScore	*p*	FC
238578_at	TMEM182	0.00	2.30	0.00	4.09
42361_g_at	CCHCR1	0.00	2.12	0.00	4.11
244383_at		0.00	−2.81	0.00	−5.74
243736_at		0.00	−2.50	0.00	−6.58
224686_x_at	LRRC37A2	0.00	−2.36	0.00	−4.17
227477_at	ZMYND19	0.00	2.31	0.00	3.42
37425_g_at	CCHCR1	0.00	2.36	0.00	3.45
243209_at	KCNQ4	0.00	2.36	0.00	5.26
243149_at		0.00	−2.19	0.00	−9.22
238706_at	PAPD4	0.00	−2.32	0.00	−7.83
239597_at		0.00	−2.20	0.00	−6.99
242407_at		0.00	−2.07	0.00	−4.26
238576_at	MOCOS	0.00	3.69	0.00	3.66
225657_at	NCBP2-AS2	0.00	2.21	0.00	3.28
242770_at	LOC642236	0.00	−3.00	0.00	−4.80
240052_at	ITPR1	0.00	−2.21	0.00	−6.12
31807_at	DDX49	0.00	2.05	0.00	2.42
215942_s_at	GTSE1	0.00	2.02	0.00	4.53
218586_at	MRGBP	0.00	2.33	0.00	3.94
238587_at	UBASH3B	0.00	2.43	0.00	3.95
239673_at		0.00	−2.22	0.00	−5.55
227371_at	BAIAP2L1	0.00	2.09	0.00	4.30
239802_at	SAP30L	0.00	−3.35	0.00	− 2.20
242143_at		0.00	−2.46	0.00	−5.86
32042_at	ENOX2	0.00	2.67	0.00	3.66
238462_at	UBASH3B	0.00	2.52	0.00	5.08
234788_x_at		0.00	−2.59	0.00	−4.13
243561_at		0.00	−2.13	0.00	−5.59
221906_at	TXNRD3 /// TXNRD3NB	0.00	2.21	0.00	3.23
239886_at		0.00	−2.26	0.00	−3.87
242787_at		0.00	2.45	0.00	4.98
225612_s_at	B3GNT5	0.00	2.15	0.00	8.41
219490_s_at	DCLRE1B	0.00	2.26	0.00	2.09
229551_x_at	ZNF367	0.00	2.07	0.00	4.97
218868_at	ACTR3B	0.00	3.49	0.00	5.27
223492_s_at	LRRFIP1	0.00	−3.44	0.00	−5.47
209825_s_at	MIR3658 /// UCK2	0.00	4.18	0.00	4.44
224712_x_at	SMIM7	0.00	−3.02	0.00	−2.79
220018_at	CBLL1	0.00	3.01	0.00	3.61
227484_at	SRGAP1	0.00	−2.68	0.00	−4.82
238712_at		0.00	−3.37	0.00	−5.56
232113_at	AK021804	0.00	−2.20	0.00	−6.19
239232_at	MSI2	0.00	−2.25	0.00	−5.37
243584_at		0.00	−2.37	0.00	−5.98
241472_at	DMXL1	0.00	−2.33	0.00	−4.77
238595_at		0.00	−2.14	0.00	−5.47
242769_at		0.00	−2.76	0.00	−3.64
221747_at	TNS1	0.00	−2.00	0.00	−4.15
230419_at	SOX9-AS1	0.00	2.93	0.00	3.22
238075_at	CHEK1	0.00	2.00	0.00	4.83
241102_at		0.00	−2.13	0.00	−4.32
230356_at	RP13-238F13.5	0.00	2.47	0.00	5.95
243216_x_at		0.00	−2.35	0.00	−3.68
60474_at	FERMT1	0.00	3.62	0.00	4.90
244080_at		0.00	2.74	0.00	4.48
232202_at	FAM83B	0.00	2.78	0.00	6.26
223381_at	NUF2	0.00	2.38	0.00	7.54
242572_at		0.00	−2.05	0.00	−4.05
211452_x_at	LRRFIP1	0.00	−2.06	0.00	−2.35
211501_s_at	EIF3B	0.00	2.54	0.00	2.88
222962_s_at	MCM10	0.00	2.72	0.00	5.52
220184_at	NANOG	0.00	−2.43	0.00	−4.33
229944_at	OPRK1	0.00	4.02	0.00	3.06
242403_at		0.00	−2.14	0.00	−5.03
232726_at		0.00	−2.54	0.00	−5.54
232092_at	SLC25A51	0.00	2.77	0.00	2.21
224590_at	XIST	0.00	−2.45	0.00	−6.62
205393_s_at	CHEK1	0.00	3.96	0.00	4.72
236982_at		0.00	2.88	0.00	2.62
222608_s_at	ANLN	0.00	2.51	0.00	7.18
240666_at		0.00	−2.09	0.00	−4.55
243709_at	SLC38A9	0.00	−2.03	0.00	−4.27
244842_x_at		0.00	−2.41	0.00	−2.53
212452_x_at	KAT6B	0.00	−2.04	0.00	− 2.95
231200_at	LSM14B	0.00	2.93	0.00	4.05
233037_at		0.00	−3.29	0.00	−4.71
236494_x_at		0.00	−2.34	0.00	−4.73
232889_at		0.00	−2.64	0.00	−7.07
238724_at		0.00	2.13	0.00	2.18
244535_at		0.00	−3.02	0.00	−3.50
223700_at	MND1	0.00	2.61	0.00	5.90
229865_at	FNDC3B///LOC101928615	0.00	2.46	0.00	4.63
243834_at	TNRC6A	0.00	−2.87	0.00	−3.29
241210_at		0.00	−2.59	0.00	−2.78
59631_at	TXNRD3///TXNRD3NB	0.00	2.80	0.00	3.08
242467_at		0.00	−2.84	0.00	−5.35
243170_at	AC092620.2	0.00	−2.02	0.00	−4.01
215397_x_at		0.00	−2.12	0.00	−3.55
211594_s_at	MRPL9	0.00	2.20	0.00	3.03
232271_at	HNF4G	0.00	2.62	0.00	2.50
225533_at	PHF19	0.00	3.14	0.00	2.43
226320_at	ALYREF	0.00	2.90	0.00	4.04
238969_at	C3orf55	0.00	2.29	0.00	3.36
222358_x_at		0.00	−2.30	0.00	−2.69
223038_s_at	FAM60A	0.00	2.38	0.00	4.93
223784_at	TMEM27	0.00	3.22	0.00	3.64
239576_at	MTUS1	0.00	−2.41	0.00	−3.82
207534_at	MAGEB1	0.00	2.04	0.00	2.05
243039_at		0.00	−2.24	0.00	−3.65
241457_at		0.00	−2.64	0.00	−7.47

### KEGG pathway enrichment and GO functional enrichment analyses

**Fig. 3:**
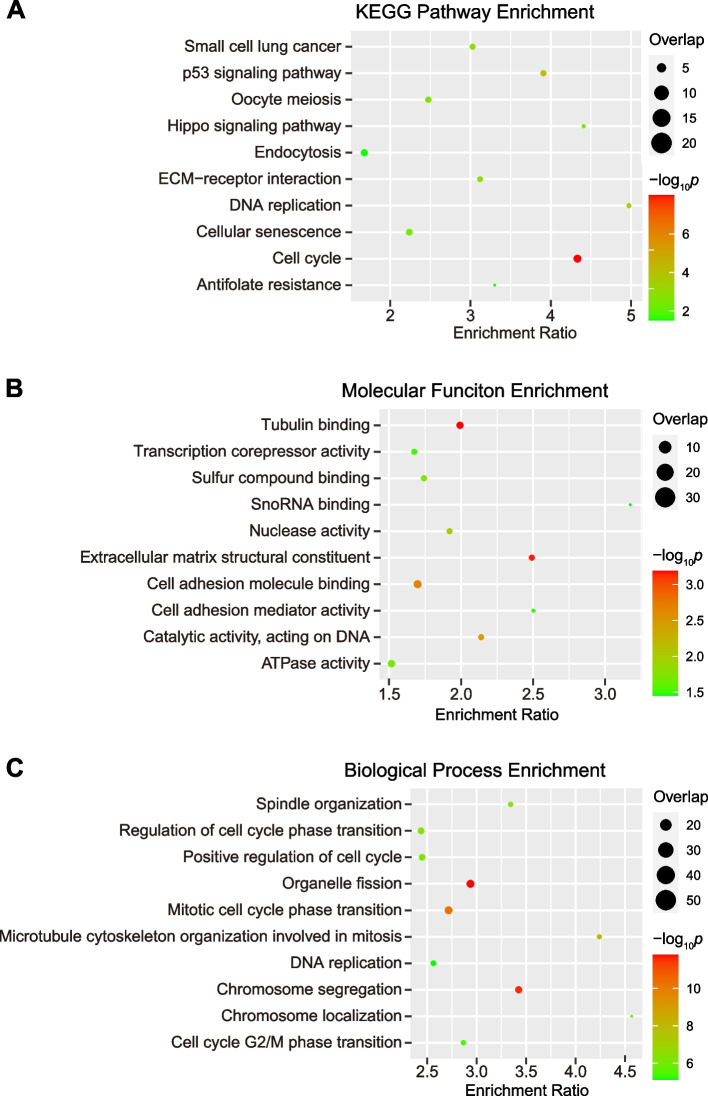
KEGG pathways and GO terms of the DEGs. **A** KEGG pathways, **B** molecular function category, **C** GO biological process category. The color of each circle indicates the significance of the enrichment, with colors closer to red representing smaller *p*-values. The size of each circle corresponds to the number of DEGs enriched in that term, with larger circles indicating a higher number of DEGs

We conducted KEGG pathway enrichment analysis and gene ontology (GO) analysis using WebGestalt to ascertain the significant biological roles and molecular functions associated with the identified DEGs. As a result, we observed several enriched biological processes, including the p53 signaling pathway, extracellular matrix (ECM) structural constituents, cell cycle, and antifolate resistance (Fig. 3, full list in Table S2). Among the significantly enriched biological processes, the top overrepresented groups were related to the regulation of cell differentiation, proliferation, migration, and the cell cycle (Fig. 3, full list in Table S4).

### Characterization of proteins encoded by the DEGs according to PPI network analysis

**Fig. 4:**
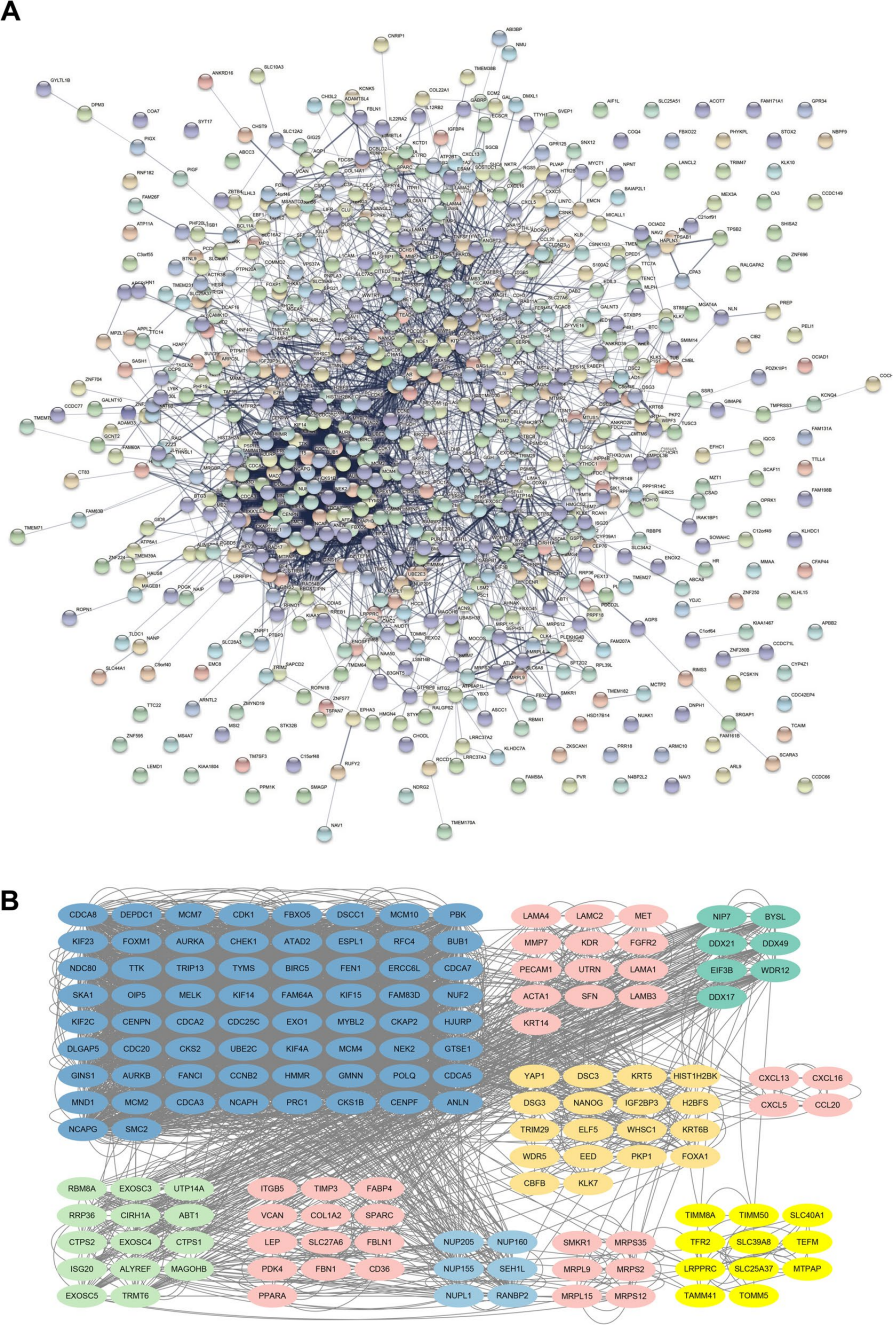
Analysis potential interactions of DEGs by PPI networks. **A** PPI networks of the 762 confirmed DEGs. **B** PPI networks of the DEGs related to top 10 clusters. DEGs with the same color represent those grouped within the same cluster

To gain further insight into the biological characteristics of the proteins encoded by the identified DEGs, we performed a protein–protein interaction (PPI) network analysis using STRING. The PPI network revealed a complex network of interactions among the DEGs (Fig. 4A). We simplified the network and identified highly interconnected regions by clustering the network using the MCODE algorithm. We present the top 10 subnetworks generated from this analysis in Fig. 4B and Table 3. We also performed further KEGG pathway enrichment analyses within these subnetworks (Table 4). These enrichment results showed that the first cluster was significantly associated with the cell cycle pathway. It is also involved in oocyte meiosis and progesterone-mediated oocyte maturation—all of which are menopause-related pathways. The second cluster was significantly associated with several tumor signaling pathways, such as the PI3K-Akt signaling pathway, EGFR tyrosine kinase inhibitor resistance pathway, Ras signaling pathway, and MAPK signaling pathway.

**Table 3:** Subnetworks in PPI network

Cluster	Score	Nodes	Edges	Genes
1	57.552	66	3856	FAM83D, MCM2, GINS1, CDC25C, POLQ, BUB1, TYMS, MCM4, CDK1, CDCA2, NDC80, CDCA5, KIF14, KIF23, SMC2, KIF4A, NCAPG, CHEK1, CDCA7, PRC1, FAM64A, CENPF, FANCI, PBK, DLGAP5, CKS2, AURKB, MND1, ATAD2, ESPL1, HMMR, ERCC6L, GMNN, KIF2C, CKAP2, FBXO5, MELK, MCM10, OIP5, CDCA8, RFC4, MYBL2, CCNB2, AURKA, CDC20, CENPN, TRIP13, UBE2C, ANLN, SKA1, TTK, NCAPH, NEK2, CDCA3, DSCC1, KIF15, NUF2, GTSE1, FEN1, BIRC5, EXO1, HJURP, CKS1B, DEPDC1, MCM7, FOXM1
2	5.571	13	78	LAMB3, SFN, MMP7, LAMA4, FGFR2, KRT14, UTRN, ACTA1, PECAM1, LAMC2, KDR, LAMA1, MET
3	5	7	40	WDR12, DDX17, BYSL, DDX21, NIP7, DDX49, EIF3B
4	4.947	18	94	H2BFS, HIST1H2BK, DSG3, PKP1, KRT6B, FOXA1, TRIM29, EED, WHSC1, KRT5, DSC3, CBFB, KLK7, WDR5, ELF5, YAP1, NANOG, IGF2BP3
5	4.8	14	72	RRP36, ISG20, CIRH1A, TRMT6, CTPS2, ABT1, EXOSC4, CTPS1, EXOSC3, RBM8A, EXOSC5, MAGOHB, UTP14A, ALYREF
6	4.571	13	64	CD36, FBN1, LEP, VCAN, FBLN1, ITGB5, FABP4, COL1A2, SLC27A6, PDK4, SPARC, TIMP3, PPARA
7	4.286	6	30	NUP155, RANBP2, NUP160, NUPL1, SEH1L, NUP205
8	4	6	28	MRPS35, MRPL9, SMKR1, MRPS2, MRPS12, MRPL15
9	2.4	4	12	CXCL16, CCL20, CXCL5, CXCL13
10	2.333	11	28	TIMM8A, TFR2, SLC39A8, TEFM, MTPAP, TAMM41, TOMM5, SLC40A1, LRPPRC, TIMM50, SLC25A37

**Table 4:**
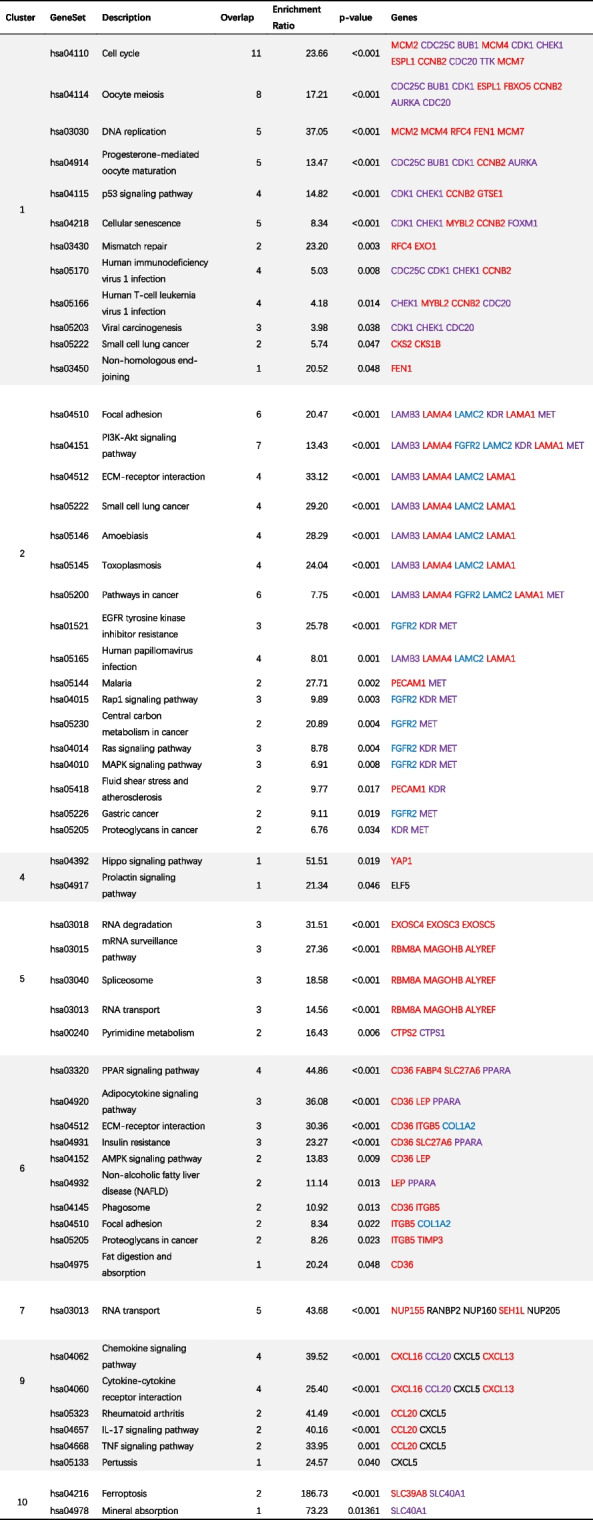
KEGG enrichment of subnetworks

Cluster 3 and 8 are not involved in any KEGG pathways. Red genes are associated with OS; Blue genes have target compounds; Purple genes are associated with OS and have target compounds

### Targeted compounds and clinical significance of DEGs

Of the 762 validated genes, 89 genes were found to have targeted compounds (Table 4; full list for DEGs in Table S5). Among these genes, 15 were derived from Cluster 1, and 7 were derived from Cluster 2. Furthermore, 73 genes with targeted compounds were related to the OS of breast cancer patients (Table 4; full list for DEGs in Table S5).

## Discussion

In this study, we investigated the differential gene expression between premenopausal and postmenopausal breast cancer patients by analyzing eight breast cancer datasets comprising 693 samples. We aimed to enhance the reliability of our analysis results by employing two different algorithms. As a result, we identified 762 DEGs that exhibited significant differences between the two groups. Among these, multiple genes have been well clarified to be associated with tumour initiation and progression. These include Matrix Metallopeptidase 7 (MMP7), transcript factors of YAP1 (one of the most important effectors of the Hippo pathway) and FOXM1, fibroblast growth factor receptor 2 (FGFR2), Eukaryotic initiation factor 3B (EIF3B), Kinesin Family Members (Kif14, Kif4A, Kif23 and Kif2C), Cyclin Dependent Kinase 1 (CDK1), Cell division cycle proteins (CDCA3, CDCA5, CDCA7, CDCA8, CDCA20 and CDC25C) and Check point Kinase 1 (CHEK1). Some of these genes have also been found to be associated with breast cancer metastasis in our previous research [25].

Among the top enriched pathways, the p53 signaling pathway and Hippo pathway are particularly remarkable, because they are involved in various intracellular regulations, including cellular senescence, energy metabolism regulating and blocking metastasis. The p53 signaling pathway, crucial in tumorigenesis [26], is frequently mutated in various human tumors, leading to a loss of its inhibitory effect on tumor growth. In this report, CDKN2A, a gene within the p53 pathway, is involved in p53-dependent cellular senescence, proliferation, and apoptosis, while it may be a pioneering prognostic predictor for breast cancer [27, 28]. Furthermore, Cyclin D1 phosphorylates Rb by binding to cyclin-dependent kinase (CDK) 4/6, resulting in activation of E2F transcription and cell cycle transition from her G1 phase to S phase. The tumor tumor-suppressive role of SERPINB5 in breast cancer is also supported by experimental evidence [29]. On the other hand, the Hippo pathway, originally discovered in *Drosophila melanogaster* as a crucial regulator of tissue development, is involved in tumorigenesis by regulating cell proliferation and apoptosis. For example, aberrations in the Hippo pathway and YAP/TAZ-TEAD activity are closely related to various human cancers, while targeting the Hippo pathway for treatment remains a compelling challenge [30].

Of particular interest, four genes (TYMS, GART, ABCC3, and GGH) were notably found to be associated with folate metabolism and involved in antifolate resistance. To date, antifolates targeting folate metabolism have played a crucial role in the treatment of malignant tumors. Various antifolates, such as the 4-amino folic acid analogue aminopterin, its homologue 4-amino-10-methylfolic acid (methotrexate), raltitrexed (Tomudex; ZD1694), and pemetrexed (Alimta; MTA, LY231514), have been discovered and introduced into oncology clinics for the chemotherapeutic treatment of childhood acute lymphoblastic leukemia, colorectal cancer, malignant pleural mesothelioma, and non-small cell lung cancer [31–35].

Raltitrexed and pemetrexed selectively inhibit glycinamide ribonucleotide transformylase (GART) and thymidylate synthase (TYMS), which are crucial for the de novo biosynthesis of purine and thymidine nucleotides, respectively. These antifolates have been introduced for the treatment of malignant tumors. ATP-binding cassette sub-family C member 3 (ABCC3, also known as MRP3), a member of the ATP-driven multidrug resistance (MDR) transporters, mediates the efflux of folates and hydrophilic antifolates. Gamma-glutamyl hydrolase (GGH) catalyzes the removal of gamma-linked polyglutamates from (anti)folylpolygamma-glutamates. Additionally, a recent study has shown that the expression level of GGH is associated with poor prognosis and unfavorable clinical outcomes in invasive breast cancer [36]. We believe that the association between these four genes and antifolates represents one of multiple pathways that could potentially act in both premenopausal and postmenopausal breast cancer.

Further KEGG pathway enrichment analysis based on the PPI subnetwork provided additional information. The first cluster was significantly associated with several important pathways, including the cell cycle, oocyte meiosis, and progesterone-mediated oocyte maturation pathways. The cell cycle is fundamental to the growth and development of all organisms and plays a significant role in cancer development and progression. For example, dysregulation of the cell cycle is a hallmark of cancer, and many chemotherapeutic drugs exert their effects by targeting the cell cycle machinery [37]. We identified several DEGs involved in the cell cycle, including CDK1, CHEK1, CDC25C, BUB1, CDC20, and TTK, which not only are related to breast cancer patient survival but also have existing targeted drugs. However, none have been reported in association with menopause. How these genes affect premenopausal and postmenopausal breast cancer has not yet been fully demonstrated. Further study of these genes related to the cell cycle pathway will help us understand the mechanism of breast cancer for different menopausal statuses and strengthen the potential utility of these genes as therapeutic targets. In addition, CDK1 and CHEK1 are involved in the p53 signaling pathway, indicating the potential effect of menopausal status on the activity of p53 signaling.

Consistent with the key role of menopause in our study, we observed that DEGs involved in oocyte meiosis and progesterone-mediated oocyte maturation, two pathways closely associated with reproductive aging and cessation, also emerged as significant in our analysis. It is widely accepted that women’s hormonal milieu undergoes significant changes during menopause, with potential implications for breast cancer biology [38]. Previous studies have reported the association of these pathways with breast cancer [39–41]. In addition to CDC25C, BUB1, and CDK1 mentioned above, AURKA, which plays a role in both pathways, is linked to survival and has targeted drugs. Importantly, AURKA has been found to be associated with an increased risk of invasive breast cancer among postmenopausal women [42].

The second cluster of DEGs, including FGFR2, KDR2 and MET, indicates the importance of key cancer-related pathways, including the PI3K-Akt signaling pathway, EGFR tyrosine kinase inhibitor resistance pathway, Rap1 signaling pathway, Ras signaling pathway, and MAPK signaling pathway. A few studies have reported associations of these pathways with breast cancer. In addition, drugs targeting these signaling pathways are available. For the first time, our study reveals a connection between these signaling pathways and menopausal status, laying the groundwork for future clinical development of breast cancer treatment strategies that cater to women with different menopausal statuses. Among these DEGs, KDR and MET are linked to survival and have available targeted drugs. Therapies targeting these key genes may be effective in improving patient outcomes. Additionally, one GWAS presented solid evidence of a strong association between the FGFR2 locus and ER status in breast cancer patients [43]. Another study found that menopause has a greater impact on ER- than ER+ breast cancer incidence [44]. These findings, along with ours, hint at the relationship between breast cancer, menopausal status, and ER status.

Interestingly, the Cluster 6 genes involved in PPAR signaling and adipose metabolism showed different expression between premenopausal and postmenopausal breast cancer patients. It has been well established that after menopause, lower levels of estrogen can lead to the accumulation of fat around the waist instead of the hips and thighs. For postmenopausal women, abdominal fat makes up 15 to 20% of their total body weight, compared to 5 to 8% in premenopausal women [45]. This also validates the reliability of our differential expression analysis results. Notably, adiposity is a risk factor for developing breast cancer in postmenopausal women, as breast fat has a major role in the genesis and progression of breast cancer. Rose et al. argued that obese postmenopausal women have an increased breast cancer risk, the principal mechanism for which is elevated estrogen production by adipose tissue [46]. Our analysis showed that DEGs (CD36, FABP4, SLC27A6, PPARA) enriched in the PPAR signaling pathway were all strongly associated with patient survival. However, whether menopause-associated obesity affects the initiation and progression of breast cancer remains an open question.

Additionally, many chemokines or cytokines, such as CCL20, CXCL5, and CXCL13 (Cluster 9), had significantly different expression levels between the two populations, which indicates differences in the tumor microenvironment. This difference could lead to a change in the infiltration of immune cells in tumor tissues and affect the efficacy of immune treatment. Locally produced and systemic cytokines are likely to affect breast cancer growth and behavior [47].

Compared with previous studies, our research benefits from a larger sample size and the use of two different algorithms to enhance the robustness of the results. In addition, the MAMA algorithm allows us to analyze data from different geographic regions. The studies included in our analysis encompass samples not just from the United States but also from Germany, France, and Belgium. This geographical diversity ensures a more global representation. However, this study has several limitations. First, some subsets lacked crucial clinical information, preventing us from analyzing the effect of clinical factors on gene expression across the entire cohort, even though we understand that some clinical factors, such as age and race, might affect menopausal status or gene expression. Second, despite using two algorithms to bolster the robustness of our results, it was challenging to determine whether we overlooked an essential gene due to algorithm differences. Third, it would be preferable to have an independent validation set. Therefore, we are attempting to collect our own clinical samples and pay more attention to these points mentioned above in our future studies. Other databases, such as TCGA, are also valuable resources for cancer research [48–50], but we did not use them in this study because they did not meet the requirements of the MAMA algorithm.

In conclusion, we utilized two differential expression analysis methods to identify several DEGs associated with menopausal status in a large integrated cohort. The interactions of the DEGs were depicted through PPI networks. Furthermore, we identified several key pathways. Most of our results related to menopausal status are reported for the first time; thus, these findings could provide a valuable reference for treating patients with premenopausal and postmenopausal breast cancer. Understanding the DEGs between premenopausal and postmenopausal breast cancer and elucidating their roles in the development and progression of the disease can offer valuable insights into its underlying mechanisms. Further studies are needed to comprehensively investigate this relationship and uncover the specific mechanisms involved. Continued research in this area will help improve our understanding of breast cancer and potentially lead to the development of more effective treatments tailored to the specific needs of premenopausal and postmenopausal patients.

### Supplementary Information

**Additional file 1: Supplementary Table 1.** *p*-value and fold change of each gene. **Supplemetary Table 2.** KEGG enrichment of DEGs. **Supplemetary Table 3.** Molecular funciton enrichement of DEGs. **Supplemetary Table 4.** Biological process enrichement of DEGs (Top 50). **Supplemetary Table 5.** Survival and Target Compounds of DEGss.

## Data Availability

The datasets used or analyzed during the present study are available from the corresponding author on reasonable request. The direct links to the data obtained from GEO database: GSE76124: https://www.ncbi.nlm.nih.gov/geo/query/acc.cgi?acc=GSE76124 GSE50948: https://www.ncbi.nlm.nih.gov/geo/query/acc.cgi?acc=GSE50948 GSE71258: https://www.ncbi.nlm.nih.gov/geo/query/acc.cgi?acc=GSE71258 GSE43365: https://www.ncbi.nlm.nih.gov/geo/query/acc.cgi?acc=GSE43365 GSE76274: https://www.ncbi.nlm.nih.gov/geo/query/acc.cgi?acc=GSE76274 GSE58792: https://www.ncbi.nlm.nih.gov/geo/query/acc.cgi?acc=GSE58792 GSE43502: https://www.ncbi.nlm.nih.gov/geo/query/acc.cgi?acc=GSE43502 GSE140494: https://www.ncbi.nlm.nih.gov/geo/query/acc.cgi?acc=GSE140494

## References

[1] Sung H, Ferlay J, Siegel RL (2021). Global Cancer statistics 2020: GLOBOCAN estimates of incidence and mortality worldwide for 36 cancers in 185 countries. CA Cancer J Clin.

[2] Brisken C, O'Malley B (2010). Hormone action in the mammary gland. Cold Spring Harb Perspect Biol.

[3] Manson JE, Aragaki AK, Rossouw JE (2017). Menopausal hormone therapy and Long-term all-cause and cause-specific mortality: the Women's Health Initiative randomized trials. JAMA.

[4] Heer E, Harper A, Escandor N, Sung H, McCormack V, Fidler-Benaoudia MM (2020). Global burden and trends in premenopausal and postmenopausal breast cancer: a population-based study. Lancet Glob Health.

[5] Beral V, Peto R, Pirie K, Reeves G (2019). Menopausal hormone therapy and 20-year breast cancer mortality. Lancet.

[6] Collaborative Group on Hormonal Factors in Breast C (2019). Type and timing of menopausal hormone therapy and breast cancer risk: individual participant meta-analysis of the worldwide epidemiological evidence. Lancet.

[7] Irizarry RA, Hobbs B, Collin F (2003). Exploration, normalization, and summaries of high density oligonucleotide array probe level data. Biostatistics.

[8] Zhang Z, Fenstermacher D (2005). An introduction to MAMA (Meta-analysis of MicroArray data) system. Conf Proc IEEE Eng Med Biol Soc.

[9] Ritchie ME, Phipson B, Wu D (2015). Limma powers differential expression analyses for RNA-sequencing and microarray studies. Nucleic Acids Res.

[10] Liao Y, Wang J, Jaehnig EJ, Shi Z, Zhang B (2019). WebGestalt 2019: gene set analysis toolkit with revamped UIs and APIs. Nucleic Acids Res.

[11] Kanehisa M, Goto S (2000). KEGG: Kyoto encyclopedia of genes and genomes. Nucleic Acids Res.

[12] Szklarczyk D, Gable AL, Lyon D (2019). STRING v11: protein-protein association networks with increased coverage, supporting functional discovery in genome-wide experimental datasets. Nucleic Acids Res.

[13] Shannon P, Markiel A, Ozier O (2003). Cytoscape: a software environment for integrated models of biomolecular interaction networks. Genome Res.

[14] Bader GD, Hogue CW (2003). An automated method for finding molecular complexes in large protein interaction networks. BMC Bioinformatics.

[15] Morris JH, Lotia S, Wu A (2014). setsApp for Cytoscape: set operations for Cytoscape nodes and edges. F1000Research.

[16] Wishart DS, Feunang YD, Guo AC (2018). DrugBank 5.0: a major update to the DrugBank database for 2018. Nucleic Acids Res.

[17] den Hollander P, Rawls K, Tsimelzon A (2016). Phosphatase PTP4A3 promotes triple-negative breast Cancer growth and predicts poor patient survival. Cancer Res.

[18] Metzger-Filho O, Catteau A, Michiels S (2013). Genomic grade index (GGI): feasibility in routine practice and impact on treatment decisions in early breast cancer. PLoS One.

[19] Yu KD, Zhu R, Zhan M (2013). Identification of prognosis-relevant subgroups in patients with chemoresistant triple-negative breast cancer. Clin Cancer Res.

[20] Prat A, Bianchini G, Thomas M (2014). Research-based PAM50 subtype predictor identifies higher responses and improved survival outcomes in HER2-positive breast cancer in the NOAH study. Clin Cancer Res.

[21] Shike M, Doane AS, Russo L, et al. The effects of soy supplementation on gene expression in breast cancer: a randomized placebo-controlled study. J Natl Cancer Inst. 2014;106(9):dju189. 10.1093/jnci/dju189 PMC4817128 25190728

[22] Xiang J, Hurchla MA, Fontana F (2015). CXCR4 protein epitope mimetic antagonist POL5551 disrupts metastasis and enhances chemotherapy effect in triple-negative breast Cancer. Mol Cancer Ther.

[23] Burstein MD, Tsimelzon A, Poage GM (2015). Comprehensive genomic analysis identifies novel subtypes and targets of triple-negative breast cancer. Clin Cancer Res.

[24] Edlund K, Madjar K, Lebrecht A (2021). Gene expression-based prediction of Neoadjuvant chemotherapy response in early breast Cancer: results of the prospective multicenter EXPRESSION trial. Clin Cancer Res.

[25] Wang L, Mo C, Wang L, Cheng M (2021). Identification of genes and pathways related to breast cancer metastasis in an integrated cohort. Eur J Clin Investig.

[26] Vousden KH, Lu X (2002). Live or let die: the cell's response to p53. Nat Rev Cancer.

[27] Cheung CT, Singh R, Kalra RS, Kaul SC, Wadhwa R (2014). Collaborator of ARF (CARF) regulates proliferative fate of human cells by dose-dependent regulation of DNA damage signaling. J Biol Chem.

[28] Cheng T, Wu Y, Liu Z (2022). CDKN2A-mediated molecular subtypes characterize the hallmarks of tumor microenvironment and guide precision medicine in triple-negative breast cancer. Front Immunol.

[29] Shi HY, Liang R, Templeton NS, Zhang M (2002). Inhibition of breast tumor progression by systemic delivery of the maspin gene in a syngeneic tumor model. Mol Ther.

[30] Wu J, Chen KJ (1988). Platelet ultrastructure and function of coronary heart disease in patients with the blood-stasis symptom-complex. Zhong Xi Yi Jie He Za Zhi.

[31] Allegra CJ, Chabner BA, Drake JC, Lutz R, Rodbard D, Jolivet J (1985). Enhanced inhibition of thymidylate synthase by methotrexate polyglutamates. J Biol Chem.

[32] Arkin H, Ohnuma T, Kamen BA, Holland JF, Vallabhajosula S (1989). Multidrug resistance in a human leukemic cell line selected for resistance to trimetrexate. Cancer Res.

[33] Barnes MJ, Taylor GA, Newell DR (2000). Development of a whole cell assay to measure methotrexate-induced inhibition of thymidylate synthase and de novo purine synthesis in leukaemia cells. Biochem Pharmacol.

[34] Papamichael D (2000). The use of thymidylate synthase inhibitors in the treatment of advanced colorectal cancer: current status. Stem Cells.

[35] Gonen N, Assaraf YG (2012). Antifolates in cancer therapy: structure, activity and mechanisms of drug resistance. Drug Resist Updat.

[36] Shubbar E, Helou K, Kovacs A (2013). High levels of gamma-glutamyl hydrolase (GGH) are associated with poor prognosis and unfavorable clinical outcomes in invasive breast cancer. BMC Cancer.

[37] Schwartz GK, Shah MA (2005). Targeting the cell cycle: a new approach to cancer therapy. J Clin Oncol.

[38] Marchant DJ (1982). Epidemiology of breast cancer. Clin Obstet Gynecol.

[39] Wang N, Zhang H, Li D, Jiang C, Zhao H, Teng Y (2021). Identification of novel biomarkers in breast cancer via integrated bioinformatics analysis and experimental validation. Bioengineered.

[40] Liu Z, Liang G, Tan L, Su AN, Jiang W, Gong C (2017). High-efficient screening method for identification of key genes in breast Cancer through microarray and bioinformatics. Anticancer Res.

[41] Dong LF, Xu SY, Long JP, Wan F, Chen YD (2017). RNA-sequence analysis reveals differentially expressed genes (DEGs) in patients exhibiting different risks of tumor metastasis. Med Sci Monit.

[42] Cox DG, Hankinson SE, Hunter DJ (2006). Polymorphisms of the AURKA (STK15/Aurora kinase) gene and Breast Cancer risk (United States). Cancer Causes Control.

[43] Cox DG, Curtit E, Romieu G (2016). GWAS in the SIGNAL/PHARE clinical cohort restricts the association between the FGFR2 locus and estrogen receptor status to HER2-negative breast cancer patients. Oncotarget.

[44] Tarone RE, Chu KC (2002). The greater impact of menopause on ER- than ER+ breast cancer incidence: a possible explanation (United States). Cancer Causes Control.

[45] Fenton A (2021). Weight, shape, and body composition changes at menopause. J Midlife Health.

[46] Rose DP, Gracheck PJ, Vona-Davis L (2015). The interactions of obesity, inflammation and insulin resistance in breast Cancer. Cancers.

[47] Eden JA (2013). Menopausal status, adipose tissue, and breast cancer risk: impact of estrogen replacement therapy. Horm Mol Biol Clin Investig.

[48] Yang L, Wang S, Zhang Q (2018). Clinical significance of the immune microenvironment in ovarian cancer patients. Mol Omics.

[49] Wang S, Zhang Q, Yu C, Cao Y, Zuo Y, Yang L (2021). Immune cell infiltration-based signature for prognosis and immunogenomic analysis in breast cancer. Brief Bioinform.

[50] Wang S, Xiong Y, Zhang Q, et al. Clinical significance and immunogenomic landscape analyses of the immune cell signature based prognostic model for patients with breast cancer. Brief Bioinform. 2021;22(4):bbaa311. 10.1093/bib/bbaa311 33302293

